# The Risk of Leakage and Bleeding After Using the Ezisurg Endostapler Technique for Bariatric Surgery

**DOI:** 10.7759/cureus.75695

**Published:** 2024-12-14

**Authors:** Mohammad Alhroot, Ramadan Hassanat, Alaa Albadaina, Qasem Alqaisi, Ashraf Altamimi, Mohammad Aldaileh, Yousef Alsardia, Majed Alqaisi, Eyad Rawashdeh, Abdallah Al-Shawabkeh

**Affiliations:** 1 Department of Surgery, King Hussein Medical Hospital, Amman, JOR; 2 Department of General Surgery, Jordanian Royal Medical Services, Amman, JOR

**Keywords:** bleeding, ezisurg endostapler, laparoscopic bariatric surgery, leakage, postoperative complications

## Abstract

Background: Obesity is a growing global health issue, with a prevalence rate of 28.8% in Jordan. Bariatric surgery is the most effective treatment for morbid obesity, yet complications such as postoperative bleeding and leakage remain significant concerns. This study evaluates the safety and effectiveness of the Ezisurg endostapler (Ezisurg Medical, Shanghai, China) in laparoscopic bariatric surgery by assessing the prevalence of these complications.

Methods: A retrospective, single-center study was conducted at King Hussein Medical Hospital, Amman, Jordan. Records of 400 consecutive patients who underwent laparoscopic bariatric surgery between September 2023 and August 2024 were reviewed. Intraoperative assessment of leakage was conducted using CT scans with methylene blue test, while postoperative leakage was evaluated through Gastrografin testing and abdominal CT scans. Bleeding was assessed according to the Bleeding Academic Research Consortium (BARC) criteria. Data on patient demographics, procedure types, intraoperative and postoperative complications, and outcomes were systematically collected. Qualitative variables were summarized through frequencies and percentages, while quantitative variables were described with measures of median and interquartile range (IQR). A p-value of <0.05 was considered statistically significant.

Results: A total of 400 patients, predominantly women (80%), with a median age of 34.0 years and a median body mass index (BMI) of 43.0 kg/m² were included. Around two-thirds of patients underwent laparoscopic sleeve gastrectomy (LSG), and the other third underwent laparoscopic Roux-en-Y gastric bypass (LRYGB). The median operation time was 96 minutes. Postoperative complications occurred in 10 patients (3.6%), including bleeding in five patients (1.25%) and leakage in three patients (0.75%). Five (1.25%) patients had postoperative bleeding, of which two were treated conservatively and three required surgical reoperation, two at the trocar site and one at the stapler line.

Conclusions: The Ezisurg endostapler showed low rates of postoperative bleeding (1.25%) and leakage (0.75%), highlighting its safety and effectiveness in laparoscopic bariatric surgery. Multicenter studies and long-term follow-ups are recommended to confirm these findings, evaluate cost-effectiveness, and explore patient-specific risk factors to further improve outcomes.

## Introduction

Recently, there has been a significant rise in the prevalence rate of obesity in the Middle Eastern region, which makes obesity a global health problem. Obesity has been shown to be a risk factor for various diseases associated with adverse health outcomes [1,2]. This raises a challenge for both individuals and healthcare sectors due to the potential pathologies associated with obesity such as hypertension, cardiovascular diseases, diabetes mellitus, dyslipidemia, or even mortality [3]. In Jordan, the prevalence of obesity based on previous studies was 28.8% according to the World Health Organization (WHO) definition of obesity [4,5].

Bariatric surgery is the most effective treatment for morbid obesity with over 300,000 operations being performed in 2022 globally [6]. It is recommended for individuals with a body mass index (BMI) over 35 kg/m^2^ to consider bariatric surgery whether there are related comorbidities or not [7]. Among the various techniques of bariatric surgeries, laparoscopic Roux-en-Y gastric bypass (LRYGB) and laparoscopic sleeve gastrectomy (LSG) stand out as the most widely accepted and extensively studied procedures [8]. While the safety and efficacy of bariatric surgery are well-established, complications still arise. Common postoperative issues include staple line leaks and bleeding, which may necessitate reoperation. Leaks are the leading cause of significant postoperative morbidity and can result from mechanical stress on the staple line, infection, impaired wound healing, and localized ischemia [9,10]. Evaluating bleeding is complex and requires consideration of several factors, including the total volume of blood loss, the rate at which blood is lost, the hemodilution effects of fluids, the impact of transfusions, and the different hemodynamic and ischemic consequences. To standardize the definition of bleeding, the Bleeding Academic Research Consortium (BARC) consisting of representatives from academic research organizations, the US Food and Drug Administration (FDA), and the industry and other experts in cardiovascular disease proposed a new hierarchically graded classification in 2011 [11,12]. Bleeding is classified into the following groups: type 1 involving non-actionable bleeding and not requiring medical intervention, type 2 representing actionable bleeding requiring non-surgical medical intervention or hospitalization, types 3a and 3b representing significant bleeding with hemoglobin drops (3-5 g/dL and ≥5 g/dL, respectively), type 3c representing intracranial hemorrhage, type 4 involving severe perioperative bleeding, and types 5a and 5b representing probable and definite fatal bleeding, respectively [13].

Bariatric surgeries have shown recent advancements in including laparoscopic techniques, making them safer, more cost-effective, and reversible alternatives to traditional bariatric procedures [14]. Laparoscopic bariatric treatments offer greater weight loss effects than medication but less than conventional bariatric surgery; however, they have been shown to have lower complication rates compared to surgery [15]. Endoscopic linear cutter staplers are essential instruments in minimally invasive surgery, especially in gastrointestinal, thoracic, and bariatric surgeries, guaranteeing secure tissue closure and enhancing patient outcomes. The Ezisurg endostapler (Ezisurg Medical, Shanghai, China) provides 120 articulation which allows a fully vertical cutting and suturing, which reduces the risk of postoperative leakage. The linear cutting stapler and its loading units are designed for the transection, resection, and creation of anastomoses and were approved by the FDA in 2021. They feature two triple-staggered rows of titanium staples, allowing for simultaneous tissue division along a central line. It is offered in three lengths, 260 mm, 350 mm, and 440 mm, and five sizes to accommodate different tissue thicknesses: 2 mm, 2.5 mm, 3.5 mm, 3.8 mm, and 4.1 mm. The device can be reloaded and fired up to 13 times during a single surgical procedure [16,17].

In this retrospective single-center study, we aim to assess the prevalence of postoperative bleeding and leakage rates among patients undergoing laparoscopic bariatric surgery using the Ezisurg endostapler in Jordan.

## Materials and methods

Study design

This study was a retrospective, single-center, comparative analysis conducted at the general surgery unit of King Hussein Medical Hospital, Amman, Jordan. We reviewed the medical records of 400 consecutive patients who underwent bariatric surgery at the facility between September 1, 2023, and August 30, 2024. Patients with incomplete medical records or those converted to open surgery were excluded. Ethical approval for this study was obtained from the Ethical Committee of King Hussein Medical Hospital prior to data collection (approval number: 68-11-2024). Patients with crucial missing data were excluded from the study.

Data collection

Patient data were retrieved retrospectively from the hospital's electronic database. Preoperative data collected included age, sex, BMI, and procedure type. All procedures were performed by a single surgical team using the Ezisurg endostapler. In patients who underwent LSG, four to six Ezisurg staplers with 60-mm-length cartridges were used, while in patients who had LRYGB, six to eight Ezisurg staplers with 60-mm-length cartridges were used.

Intraoperative assessment of leakage was conducted using CT scans with methylene blue test, while postoperative leakage was evaluated through Gastrografin testing and abdominal CT scans. Patients with tachycardia (pulse rate >100 beats per minute) were assessed for bleeding and were considered positive with a significant drop in hemoglobin for more than 3-5 g/dL according to BARC criteria. Patients with postoperative leakage were managed with laparoscopic gastroesophageal stenting or conversion to gastric bypass if necessary. For those experiencing postoperative bleeding, some were treated conservatively, while others required surgical reoperation, targeting the trocar site or the stapler line. Vital signs and packed cell volume (PCV) levels were monitored postoperatively three times every 12 hours for patients with postoperative bleeding. Additional data on comorbidities, presence of gastroesophageal reflux disease (GERD), drainage during surgery, mean postoperative pain scores (using the Numeric Pain System (NPS)), time to first ambulation postoperatively, and hospital stay duration were also recorded.

Statistical analysis

Data were analyzed utilizing IBM SPSS Statistics for Windows, Version 26.0 (Released 2019; IBM Corp., Armonk, New York, United States). Qualitative variables were summarized through frequencies and percentages, while quantitative variables were described with measures of median and interquartile range (IQR) as they deviated from a normal distribution based on the Shapiro-Wilk test. The association between surgery type and clinical outcomes was assessed using the chi-squared test and the Wilcoxon rank-sum test, with a p-value of <0.05 considered statistically significant.

## Results

Our cohort included a total of 400 patients who underwent bariatric surgery using the Ezisurg endostapler. There was a female predominance in our cohort accounting for 225 (80%) patients, and the median cohort BMI was 43 (40, 48) kg/m^2^ (Table 1). Patients underwent either LRYGB (n=135, 33.75%) or LSG (n=265, 66.25%). Medical comorbidities included diabetes mellitus in 72 (26%) patients, hypertension in 59 (21%) patients, and GERD in 40 (14%) patients. The median preoperative laboratory values for PCV and white blood cell (WBC) were 39% (36%, 42%) and 10 (8, 12.1)×10^9^ cells/L, respectively.

**Table 1:** Baseline demographics and preoperative characteristics of the included patients. BMI: body mass index; PCV: packed cell volume; WBC: white blood cell; LRYGB: laparoscopic Roux-en-Y gastric bypass; LSG: laparoscopic sleeve gastrectomy; DM: diabetes mellitus; GERD: gastroesophageal reflux disease

Characteristic	N=400
Age (years), median (Q1, Q3)	34 (27, 41)
Age at operation (years), median (Q1, Q3)	33 (26, 40)
Sex, n (%)	
Female	225 (80%)
Male	55 (20%)
BMI (kg/m^2^), median (Q1, Q3)	43 (40, 48)
Pre-PCV, median (Q1, Q3)	39 (36, 42)
Pre-WBC, median (Q1, Q3)	10.0 (8, 12.1)
Surgery, n (%)	
LRYGB	135 (33.75%)
LSG	265 (66.3%)
DM, n (%)	72 (26%)
Hypertension, n (%)	59 (21%)
GERD, n (%)	40 (14%)

**Figure 1:**
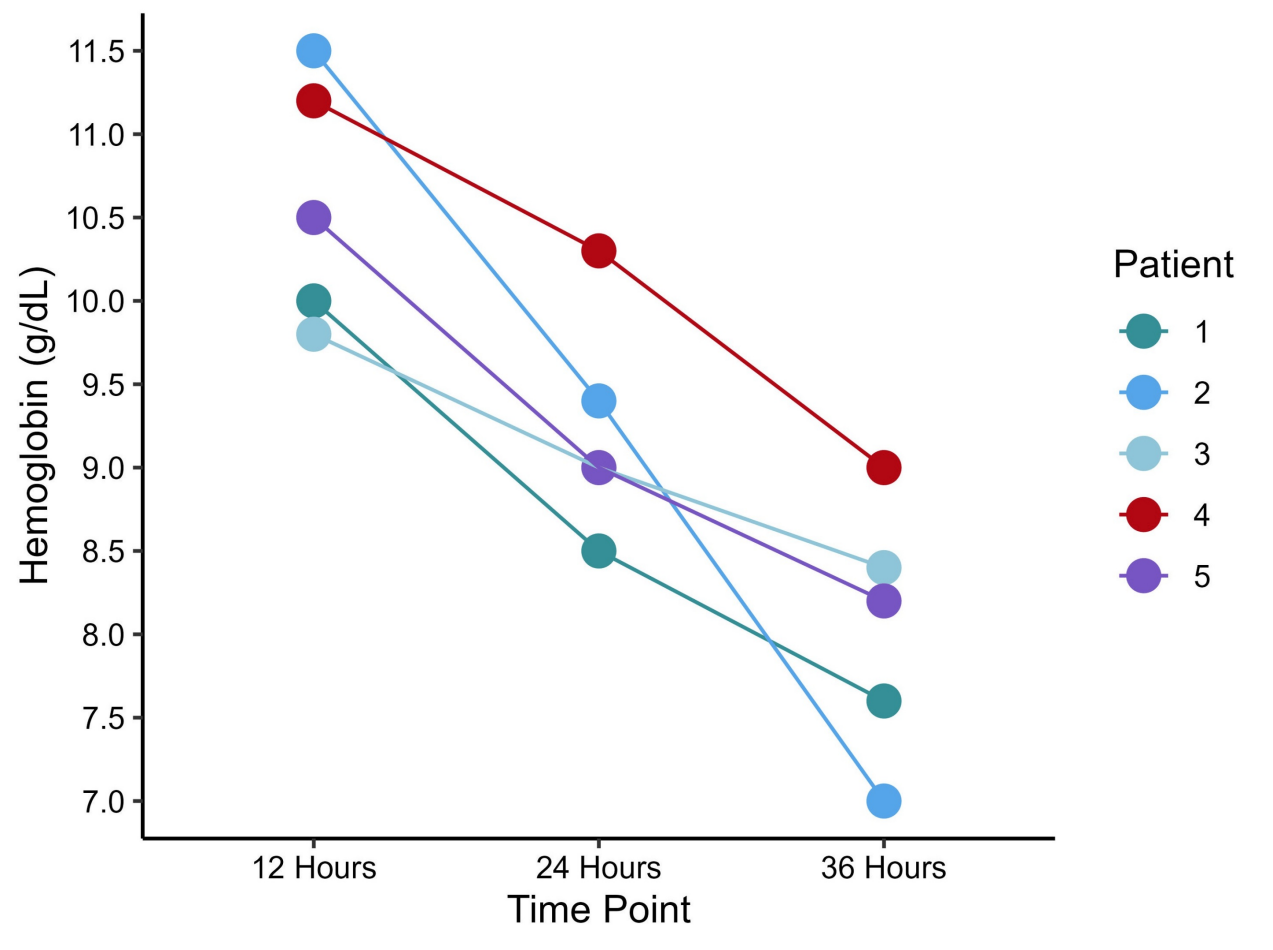
Dot plot representing hemoglobin levels in patients with postoperative bleeding.

Postoperative characteristics are presented in Table 2. Complications occurred in 10 (3.6%) patients, with three (0.75%) having postoperative leakage of which two were treated with laparoscopic gastroesophageal stenting and one patient was converted to gastric bypass. Five (1.25%) patients had postoperative bleeding (Figure 1), of which two were treated conservatively and three required surgical reoperation two at the trocar site and one at the stapler line. Median postoperative PCV and WBC values are 38% (35%, 42%) and 12 (10, 15)×10^9^ cells/L, respectively. The median operation time was 96 (78, 134) minutes. All five patients with bleeding have received blood transfusion, and the median pain score was 3.85 (2.78, 5.57).

**Table 2:** Postoperative characteristics of the included patients. PCV: packed cell volume; WBC: white blood cell

Characteristic	N=400
Post-PCV, median (Q1, Q3)	38 (35, 42)
Post-WBC, median (Q1, Q3)	12 (10, 15)
Drains, n (%)	225 (80%)
Complications, n (%)	10 (3.6%)
Operation time, median (Q1, Q3)	96 (78, 134)
Blood transfusion, n (%)	5 (1.25%)
Pain score, median (Q1, Q3)	3.85 (2.78, 5.57)
Days to full ambulation, median (Q1, Q3)	3.81 (2.33, 5.48)
Days of drainage, median (Q1, Q3)	4.84 (3.79, 6.34)
Leakage, n (%)	3 (0.75%)
Bleeding, n (%)	5 (1.25%)

Post hoc analysis for comparing the type of surgery with postoperative characteristics is shown in Table 3. Patients who underwent LSG had a significantly higher rate of complications (8% vs. 1.1%; p=0.005), a longer duration time (median: 146 vs. 84; p<0.001), longer hospital stay (median: 9.1 vs. 5.9; p<0.001), and higher pain score (median: 6.20 vs. 3.07; p<0.001).

**Table 3:** Post hoc comparison of clinical and surgical characteristics based on the type of bariatric surgery. PCV: packed cell volume; WBC: white blood cell; DM: diabetes mellitus; LRYGB: laparoscopic Roux-en-Y gastric bypass; LSG: laparoscopic sleeve gastrectomy; GERD: gastroesophageal reflux disease

Characteristic	LRYGB, N=180	LSG, N=100	P-value
Post-PCV, median (Q1, Q3)	38 (36, 41)	37.5 (35, 42)	0.7
Post-WBC, median (Q1, Q3)	12 (10, 15)	12 (10, 14)	0.9
Drains, n (%)	150 (83%)	75 (75%)	0.093
Complications, n (%)	2 (1.1%)	8 (8%)	0.005
DM, n (%)	37 (21%)	35 (35%)	0.008
Hypertension, n (%)	32 (18%)	27 (27%)	0.07
GERD, n (%)	21 (12%)	19 (19%)	0.093
Operation time, median (Q1, Q3)	84 (72, 94)	146 (132, 160)	<0.001
Blood transfusion, median (Q1, Q3)	4 (2.2%)	7 (7%)	0.059
Hospital stay, median (Q1, Q3)	5.9 (3.7, 8.6)	9.1 (6.7, 12.5)	<0.001
Pain score, median (Q1, Q3)	3.07 (2.45, 3.81)	6.20 (5.31, 7.00)	<0.001
Leak, n (%)	0 (0%)	2 (2%)	0.13
Bleeding, n (%)	0 (0%)	1 (1%)	0.4

## Discussion

Obesity is rising rapidly in the Middle East, with a reported prevalence rate of 28.8% in Jordan based on the WHO criteria for obesity [4,5]. Bariatric surgery is recognized as the most effective treatment for morbid obesity, particularly LRYGB and LSG, though both can have complications like postoperative leakage and bleeding. Recent advances in laparoscopic bariatric techniques offer a safer approach with fewer postoperative complications [9]. In this retrospective study, we assessed the bleeding and leakage rates post-laparoscopic bariatric surgery using the Ezisurg stapler in Jordan.

Our findings showed a postoperative bleeding rate of 1.25% seen in five patients who underwent LSG and were treated conservatively or by surgical reoperation. A study by Polese et al. investigating the efficacy of LSG showed that postoperative bleeding was seen in 7% of patients, who were at high bleeding risk preoperatively due to dialysis and antiplatelet medications; however, they were successfully managed with packed red blood cell transfusion [18]. Another study also showed that postoperative bleeding rates following endoscopic sleeve gastrectomy were significantly lower than LSG accounting for 1.1% in endoscopic sleeve gastrectomy and 2.6% in LSG [19]. Postoperative bleeding was shown to be the most common complication after RYGB [20]. Several intraoperative factors have been shown to increase the risk of postoperative bleeding in patients undergoing RYGB procedures such as using a circular stapler to create a mechanical gastrojejunal anastomosis, rather than a hand-sewn technique, and failing to support the staple line can elevate the risk of bleeding [21,22].

Previous research showed that LRYGB, though technically more complex and needs more surgical skills, provides more favorable postoperative outcomes compared to LSG [23]. In contrast, Rondelli et al. showed that LSG was associated with significantly lower postoperative complications than RYGB; however, there was no significant difference in postoperative bleeding rate between the two operations with a slightly lower rate in RYGB (1% vs. 2.1%) [24]. However, these differences can be due to the performance of complementary surgeries in 19.3% of LSG patients and 34.6% of LRYGB patients.

The rates of postoperative leakage (0.75%) were particularly low. This finding indicates a favorable safety profile for bariatric procedures performed using the Ezisurg endostapler. The rate of postoperative leakage following bariatric surgery differed between studies ranging between 0.1% and 5.6%. Variations in leakage rate might be attributed to differences in leakage definition [25,26]. A study by Alizadeh et al. showed that 938 out of 133,478 patients who underwent laparoscopic bariatric surgery had postoperative gastrointestinal leak with a rate of 0.7% and significant association in patients with hypoalbuminemia, hypertension, and sleep apnea [27]. Another study by Bashah et al. observed a postoperative leak in 17 patients out of 4250 who underwent LSG with a rate of 0.4%. In their study, diagnosis of postoperative leak was for patients who had symptoms and signs of a leak using abdominal CT scan and was confirmed through radiological evidence of oral contrast extravasation on CT scan or fluoroscopy or by identifying a fistulous opening during endoscopy [28]. Previous studies showed no difference between LSG and LRYGB in leakage rates, with no reported leaks at the gastrojejunal anastomosis while only one leak was reported at the jejuno-jejunal anastomosis. This was likely due to the hand-sewn technique, suggesting a potential need to improve or revise this approach [24]. It has been shown that anastomotic leaks after bariatric surgery most frequently occur along the staple line and are most likely to develop at the gastrojejunal anastomosis which is attributed to the limited blood supply to the gastric pouch [29,30].

Our study has several strengths. First, this is the first study to our knowledge in Jordan to assess the outcomes and prevalence of postoperative leakage and blood rates. However, our results should be interpreted carefully due to several limitations. First, the retrospective, single-center design limits the generalizability of the findings. Additionally, the lack of long-term follow-up prevents the assessment of late-onset complications and sustained outcomes. Future multicenter longitudinal studies are needed to increase the generalizability of the results and include a larger sample size. Comparisons with other emerging laparoscopic tools and techniques, as well as further evaluation of complementary procedures, could also enhance the understanding of safety profiles across different populations and settings. While our study highlights the safety and effectiveness of the Ezisurg endostapler, it is important to consider its broader clinical implications and limitations. The low complication rates could inform clinical decision-making and potentially shape guidelines for bariatric surgery. However, potential biases, such as selection bias from the single-center design and the impact of operator experience, may limit the generalizability of these findings. Future studies should include diverse populations, multiple centers, and varying surgical expertise to better evaluate the device's performance across different settings.

## Conclusions

Bariatric surgeries using the Ezisurg endostapler demonstrated low rates of postoperative bleeding and leakage, highlighting its potential to improve the safety profile of laparoscopic techniques for managing morbid obesity. These findings suggest clinical benefits, such as reduced complication-related costs and enhanced patient recovery. Incorporating advanced stapling devices like the Ezisurg endostapler may optimize bariatric surgical outcomes and streamline clinical workflows. Future multicenter studies should focus on long-term outcomes, cost-effectiveness analyses, and comparisons with alternative devices to strengthen the evidence base and inform clinical guidelines.
